# Supplementary material for: Detection of porcine enteric viruses (Kobuvirus, Mamastrovirus and Sapelovirus) in domestic pigs in Corsica, France
Source: PLoS One. 2022 Jan 14;17(1):e0260161. doi: 10.1371/journal.pone.0260161 (PMC8759673; doi:10.1371/journal.pone.0260161)
Supplement: S1 Table — (DOCX) [file pone.0260161.s001.docx]

**S1 Table. Positivity rates for the three viruses by pig farm**

| **Farm number** | PKoV | | PAstV-1 | | PSV | | Total |
| --- | --- | --- | --- | --- | --- | --- | --- |
|  | **N** | **%** | **N** | **%** | **N** | **%** |  |
| 1 | 27 | 65.9 | 0 | 0.0 | 13 | 31.7 | 41 |
| 2 | 28 | 27.2 | 13 | 12.6 | 70 | 68.0 | 103 |
| 3 | 25 | 29.4 | 3 | 3.5 | 56 | 65.9 | 85 |
| 4 | 61 | 48.8 | 3 | 2.4 | 88 | 70.4 | 125 |
| 5 | 25 | 56.8 | 11 | 25.0 | 38 | 86.4 | 44 |
| 6 | 9 | 45.0 | 1 | 5.0 | 9 | 45.0 | 20 |
| 7 | 20 | 22.5 | 1 | 1.1 | 62 | 69.7 | 89 |
| 8 | 10 | 27.8 | 0 | 0.0 | 21 | 58.3 | 36 |
| 9 | 37 | 59.7 | 4 | 6.4 | 41 | 66.1 | 62 |
| 10 | 25 | 41.0 | 9 | 14.8 | 37 | 60.7 | 61 |
| 11 | 13 | 32.5 | 8 | 20.0 | 17 | 42.5 | 40 |
| 12 | 59 | 80.8 | 25 | 34.2 | 65 | 89.0 | 73 |
| 13 | 24 | 55.8 | 0 | 0.0 | 0 | 0.0 | 43 |
| 14 | 32 | 71.1 | 0 | 0.0 | 16 | 35.6 | 45 |
| 15 | 9 | 36.0 | 0 | 0.0 | 16 | 64.0 | 25 |
| 16 | 4 | 25.0 | 0 | 0.0 | 14 | 87.5 | 16 |
