# Supplementary material for: Detection of porcine enteric viruses (Kobuvirus, Mamastrovirus and Sapelovirus) in domestic pigs in Corsica, France
Source: PLoS One. 2022 Jan 14;17(1):e0260161. doi: 10.1371/journal.pone.0260161 (PMC8759673; doi:10.1371/journal.pone.0260161)
Supplement: S2 Table — (DOCX) [file pone.0260161.s002.docx]

**S2 Table**

|  | **OVERALL** | **POSITIVES** | **MEAN MELT CURVE** | **CT MEAN** | **CT MIN-MAX** | **CT SD** |
| --- | --- | --- | --- | --- | --- | --- |
| **Sapelovirus** | **908** | **563** | **83.99** | **24.4** | **15-35** | **3.248** |
| **Kobuvirus** |  | **407** | **/** | **30.94** | **23-35** | **2.918** |
| **Astrovirus** |  | **78** | **/** | **31.82** | **26-35** | **2.390** |
